# Modification, Biological Evaluation and 3D QSAR Studies of Novel 2-(1,3-Diaryl- 4,5-Dihydro-1*H-*Pyrazol-5-yl)Phenol Derivatives as Inhibitors of B-Raf Kinase

**DOI:** 10.1371/journal.pone.0095702

**Published:** 2014-05-14

**Authors:** Yu-Shun Yang, Fei Zhang, Dan-Jie Tang, Yong-Hua Yang, Hai-Liang Zhu

**Affiliations:** 1 State Key Laboratory of Pharmaceutical Biotechnology, Nanjing University, Nanjing, P. R. China; 2 Institute of Chemical and Biomedical Science, Nanjing University, Nanjing, P. R. China; University of Pittsburgh School of Medicine, United States of America

## Abstract

A series of novel 2-(1,3-diaryl- 4,5-dihydro-1*H*-pyrazol-5-yl)phenol derivatives (**C1–C24**) have been synthesized. The B-Raf inhibitory activity and anti-proliferation activity of these compounds have been tested. Compound **C6** displayed the most potent biological activity against B-Raf^V600E^ (IC_50_ = 0.15 µM) and WM266.4 human melanoma cell line (GI_50_ = 1.75 µM), being comparable with the positive control (Vemurafenib and Erlotinib) and more potent than our previous best compounds. The docking simulation was performed to analyze the probable binding models and poses while the QSAR model was built to check the previous work as well as to introduce new directions. This work aimed at seeking more potent inhibitors as well as discussing some previous findings. As a result, the introduction of *ortho*-hydroxyl group on 4,5-dihydro-1*H*-pyrazole skeleton did reinforce the anti-tumor activity while enlarging the group on *N*-1 of pyrazoline was also helpful.

## Introduction

Cancer is continuing to act as a major problem of health all over the world, enacting the second cause of mortality [1]. Discovering new anticancer agents remains critically important in spite of the progress in medicine.

Ras-Raf-MEK-ERK serine threonine kinase cascade, which is also called ERK/MAP kinase pathway or ‘classical’ MAPK pathway, has been convinced to be important for cell proliferation and survival [2], [3]. It can be hyper-activated in up to 30% of human cancers [4]. All through the pathway, activating mutations in Raf have been observed most in 50–70% of cell lines and tumors in melanoma, then 40%–70% in thyroid cancer, 50–70% in ovarian cancer [5], [6], [7]. B-Raf is an isoform of Raf kinases. Approximately 90% of its activating mutations in cancers are valine for glutamic acid substitution (V600E, formally defined as V599E) [5], [8], [9]. This kind of mutations causes a 500-fold increase in the basal rate of MEK phosphorylation over wild-type B-Raf [10]. This kind of increase stimulates tumor growth and vascular endothelial growth factor secretion [11], [12]. Thus, B-Raf^V600E^ is indicated to be a therapeutic target for designing anticancer drugs [13].

Although Vemurafenib is considered to be the most potent B-Raf inhibitor now and has received FDA approval [14], researches of alternative skeletons are attempting to break the limitation of the fixed structure. Among inhibitors of B-Raf, SB-590885 has displayed potent inhibitory activity [15]. SB-590885 is a novel triarylimidazole derivative. The origin of its selectivity for B-Raf seems probably due to its interactions with several B-Raf amino acids and the presence of the indane-oxime. One particular interaction is formed between heterocyclic rings (both imidazole and pyridine) of SB-590885 and PHE583 of B-Raf [15]. Meanwhile, in spite of other pharmaceutical and agrochemical activities [16], [17], dihydropyrazole derivatives have been screened and convinced to be potent and selective inhibitors of B-Raf^V600E^ [18]. As for all the series in this paper, they avoid the quinoline moiety of Vemurafenib thus the corresponding side effect is eliminated radically.

**Figure 1:**
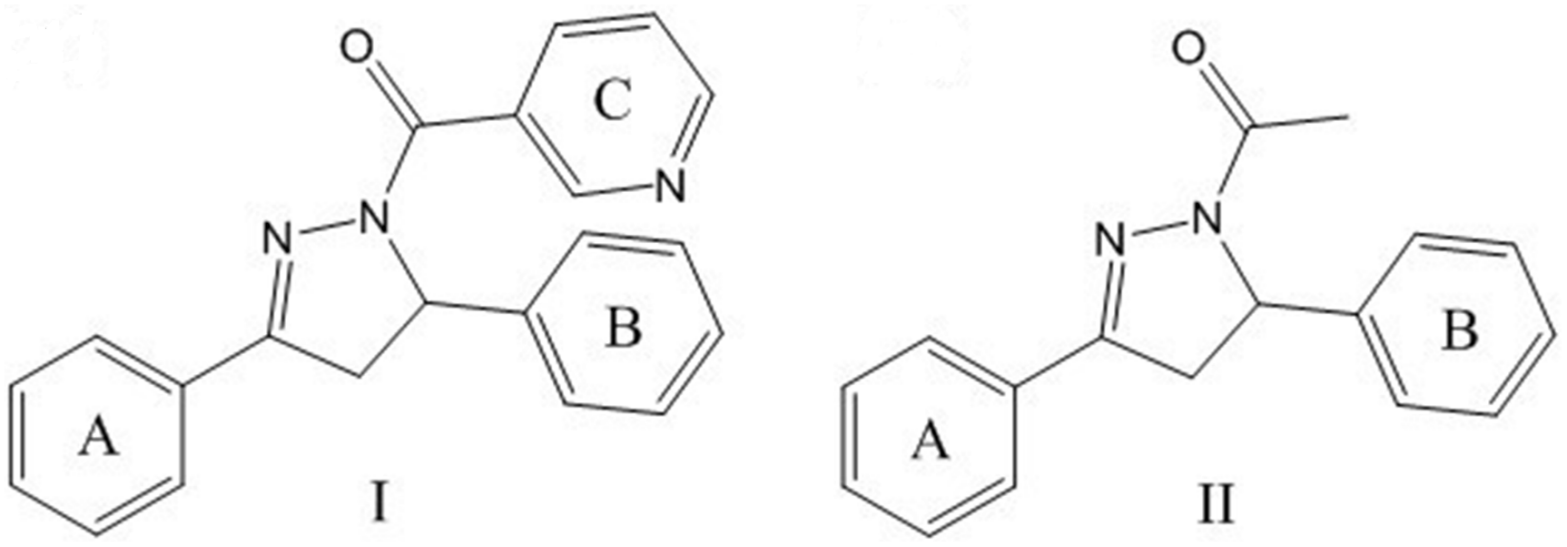
The structures of previous series I and series II.

In our previous research, a reliable 3D QSAR model was built from a series of 4,5-dihydropyrazole derivatives containing niacinamide moiety (series **I**) to visualize the SAR (Structure Activity Relationship) [19]. In that series, niacinamide moiety was relatively suitable in size. However, in another independent research (series **II**) of our group, while the niacinamide moiety was absent, inhibitory activity of the compounds was still comparable with that of the former ones [20]. The structures of both series were shown in Figure 1. Considering the reliability of the model and the structural differences, we inferred that the introduction of hydroxyl group might cause the phenomena. In this paper, we chose the skeleton of series **II** and replaced the original acetyl group with phenyl group (primarily fulfilling the requirement of size). One purpose was to check the previous model while the other was to verify the positive effect of hydroxyl group. As a preliminary exploration, the situation was simplified by defaulting the carbonyl and substitutes on the new added phenyl.

## Results and Discussion

### 1. Chemistry

**Figure 2:**
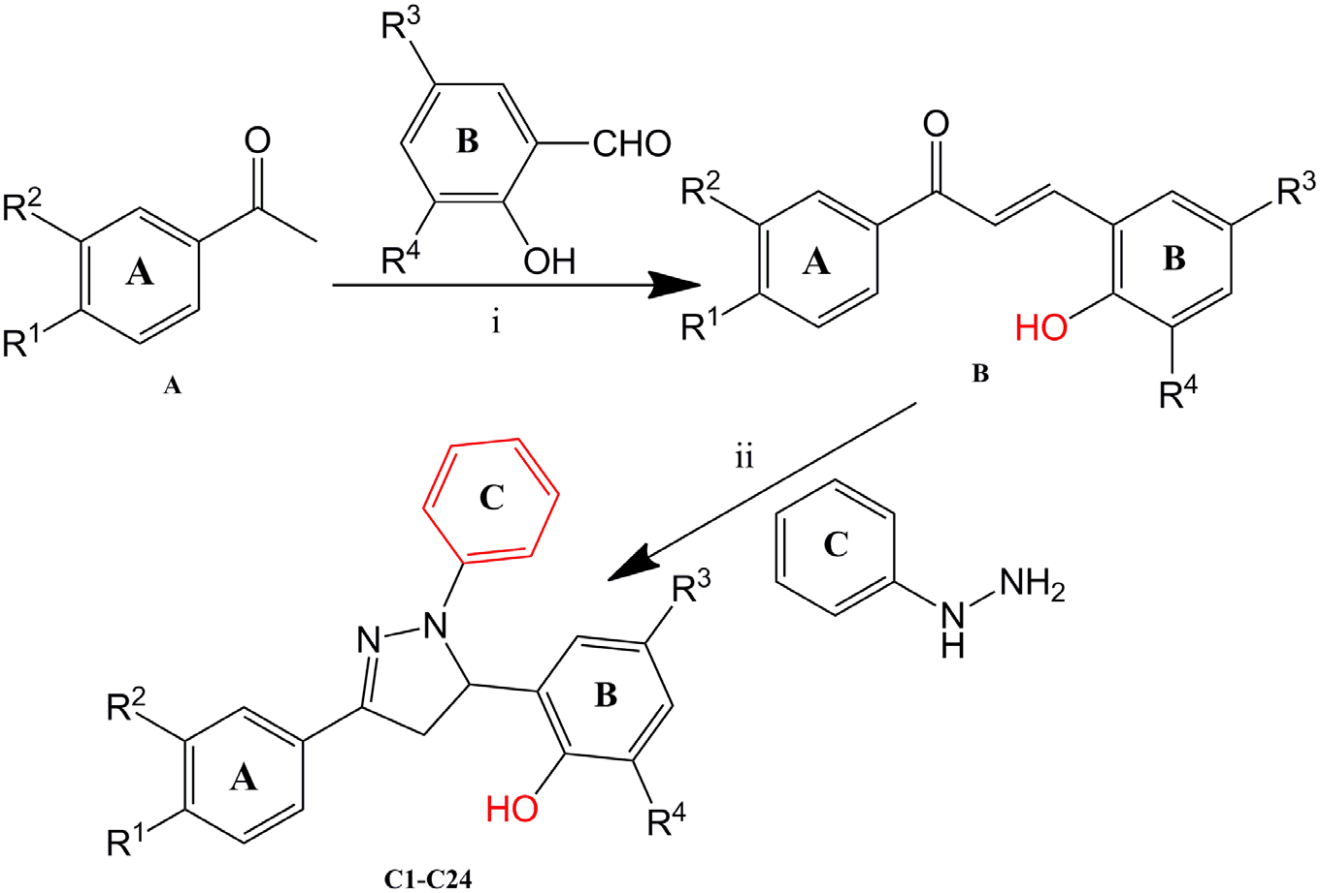
General synthesis of compounds (C1–C24). Reagents and Conditions: i) EtOH, 40% NaOH, 0°C, stir, 30 min; rt, stir, 4 h; ii) EtOH, Phenylhydrazine, 80°C, 5 h.

Twenty-four 2-(1,3-diaryl- 4,5-dihydro-1*H*-pyrazol-5-yl)phenol derivatives were synthesized and screened for their antitumor activity. All of them were synthesized for the first time except compound **C20** [21]. The general synthesis method and the structures of compounds **C1–C24** were organized in Table 1 and Figure 2. They were all prepared in two steps. Firstly, different substituted acetophenones on treatment with substituted salicylaldehyde in presence of 40% NaOH were stirred at 0°C for 30 min to avoid side reactions. Then the mixtures were placed to room temperature to continue the reaction for 4 h, yielding different analogues of chalcones (**B**). Secondly, phenylhydrazine was added to participate the cyclization of the obtained powder, leading to the corresponding target compounds **C1–C24** 2-(1,3-diaryl- 4,5-dihydro-1*H*-pyrazol-5-yl)phenol. Subsequent purification with recrystallisation was conducted and the refined compounds were finally obtained. All of the synthetic compounds gave satisfactory analytical and spectroscopic data, which were in full accordance with their depicted structures.

**Table 1:** Substitutes of the synthesized compounds.

Compound	R^1^	R^2^	R^3^	R^4^	Compound	R^1^	R^2^	R^3^	R^4^
C1	F	H	H	H	C13	F	H	Br	H
C2	Cl	H	H	H	C14	Cl	H	Br	H
C3	Br	H	H	H	C15	Br	H	Br	H
C4	CH_3_	H	H	H	C16	CH_3_	H	Br	H
C5	OCH_3_	H	H	H	C17	OCH_3_	H	Br	H
C6	Cl	Cl	H	H	C18	Cl	Cl	Br	H
C7	F	H	Cl	H	C19	F	H	Cl	Cl
C8	Cl	H	Cl	H	C20	Cl	H	Cl	Cl
C9	Br	H	Cl	H	C21	Br	H	Cl	Cl
C10	CH_3_	H	Cl	H	C22	CH_3_	H	Cl	Cl
C11	OCH_3_	H	Cl	H	C23	OCH_3_	H	Cl	Cl
C12	Cl	Cl	Cl	H	C24	Cl	Cl	Cl	Cl

### 2. Biological Activity

With a general method, all the synthesized compounds **C1–C24** were evaluated for their anti-proliferation effect and B-Raf^V600E^ inhibitory activity. The results were expressed as concentrations of IC_50_ (the half maximal inhibitory concentration of B-Raf^V600E^ mediated MEK phosphorylation) and GI_50_ (the half maximal inhibitory concentration of WM266.4 human melanoma cell line [22] growth), presented in Table 2. WM266.4 human melanoma cell line was chosen because mutations in Raf have been observed most in melanoma. Two previous best compounds **C0A** (named **27e** in previous work) [19] and **C0B** (named **3d** in previous work) [20] were taken into the same evaluation (both their test results and literature values) for comparison. As shown in Table 2, a majority of the compounds showed potent B-Raf^V600E^ inhibitory activity. It seemed that the introduction of hydroxyl group did enhance the activity while replacing original acetyl with phenyl also led to positive effect.

**Table 2:** B-Raf^V600E^ inhibitory activity and anti-proliferation activity of the synthesized compounds (**C1–C24**) as well as previous compounds **C0A** and **C0B**.

compounds	IC_50_ (*µ*M)	GI_50_ (*µ*M)	compounds	IC_50_ (*µ*M)	GI_50_ (*µ*M)
	B-Raf^V600E^	WM266.4		B-Raf^V600E^	WM266.4
C1	71.90±6.77	>50	C13	2.73±0.19	4.45±0.41
C2	0.50±0.04	2.01±0.13	C14	2.20±0.20	3.69±0.32
C3	0.63±0.06	2.09±0.18	C15	1.29±0.10	2.65±0.19
C4	1.49±0.11	2.85±0.23	C16	2.78±0.21	4.55±0.44
C5	0.51±0.05	1.98±0.17	C17	0.57±0.05	2.03±0.18
C6	0.15±0.01	1.75±0.12	C18	0.34±0.02	1.88±0.15
C7	2.60±0.23	4.26±0.36	C19	2.26±0.19	3.76±0.32
C8	2.66±0.19	4.39±0.31	C20	2.14±0.19	3.59±0.28
C9	1.37±0.13	2.73±0.21	C21	3.12±0.26	5.13±0.49
C10	3.24±0.28	5.37±0.49	C22	7.37±0.65	23.93±1.99
C11	1.07±0.08	2.43±0.16	C23	1.01±0.08	2.38±0.23
C12	0.50±0.05	1.99±0.13	C24	0.97±0.09	2.34±0.17
C0A	0.19±0.02	0.93±0.07	C0B	0.23±0.03	0.56±0.04
C0A(lit)	0.20±0.03	0.89±0.04	C0B(lit)	0.22±0.06	0.45±0.03
Erlotinib	0.06±0.01	8.12±0.75	Vemurafenib	0.03±0.005	0.21±0.02

The same as the previous researches [19], [20], the GI_50_ values of these compounds shared a similar tendency with their relevant IC_50_ values (linear regression: R square = 0.826, a normal level). This indicated the correlation between the anti-proliferative effect and the B-Raf inhibitory activity.

Out of the twenty-four compounds, **C6** displayed the most potent activity (IC_50_ = 0.15 µM; GI_50_ = 1.75 µM). The values were comparable with that of the positive control Vemurafenib (IC_50_ = 0.03 µM; GI_50_ = 0.21 µM) and Erlotinib (IC_50_ = 0.06 µM; GI_50_ = 8.12 µM). **C6** was slightly better than the previous best compounds **C0A** (IC_50_ = 0.19 µM in test; IC_50_ = 0.20 µM in literature) and obviously better than **C0B** (IC_50_ = 0.23 µM in test; IC_50_ = 0.22 µM in literature) on B-Raf inhibitory activity but less potent (**C0A**: GI_50_ = 0.93 µM in test and GI_50_ = 0.89 µM in literature; **C0B**: GI_50_ = 0.56 µM in test and GI_50_ = 0.45 µM in literature) on anti-proliferation. A possible explanation might be the influence of logP and PSA (polar surface area). With the substitutes defaulted, the skeletons of series **I** (logP = 3.359; PSA = 45.565) and series **II** (logP = 2.864; PSA = 52.901) were in better situations than that of our series (logP = 5.2; PSA = 35.83). Fortunately, it seemed that the skeleton itself displayed better B-Raf inhibitory effect, for the IC_50_ scale in this series (∼1.5 µM) was lower than that of Series **II** (∼2.3 µM). Then the disadvantage in anti-proliferation could be promoted by introducing appropriate pharmacokinetics groups.

According to the results, preliminary SAR studies were conducted to deduce the influence of structure variation on anticancer activity. Firstly, as shown, enlarging the size of acetyl enhanced the B-Raf inhibitory activity thus the previous 3D QSAR model was relatively correct. Meanwhile, the introduction of hydroxyl was helpful indeed. Secondly, we fixed ring **A** (R^1^ and R^2^) and analyzed the substitutes on ring **B** (R^3^ and R^4^). A general trend was null > bromo > chloro ≥ dichloro. For example, **C6** (IC_50_ = 0.15 µM; GI_50_ = 1.75 µM) > **C18** (IC_50_ = 0.34 µM; GI_50_ = 1.88 µM) > **C12** (IC_50_ = 0.50 µM; GI_50_ = 1.99 µM) > **C24** (IC_50_ = 0.97 µM; GI_50_ = 2.34 µM) and **C3** (IC_50_ = 0.63 µM; GI_50_ = 2.09 µM) > **C15** (IC_50_ = 1.29 µM; GI_50_ = 2.65 µM) > **C9** (IC_50_ = 1.37 µM; GI_50_ = 2.73 µM) > **C21** (IC_50_ = 3.12 µM; GI_50_ = 5.13 µM). Thus, for this point, a smaller and less negative charged substitute was preferred. The only group against this trend enjoyed a same ring **A** (*para*-fluoro). A relatively large ring **B** might be a remedy of small ring **A**. Finally, we fixed ring **B** (R^3^ and R^4^) and analyzed the substitutes on ring **A** (R^1^ and R^2^). A preliminary trend was dichloro > methoxyl ≥ bromo ≥ chloro ≥ fluoro > methyl. For example, **C18** (IC_50_ = 0.34 µM; GI_50_ = 1.88 µM) > **C17** (IC_50_ = 0.57 µM; GI_50_ = 2.03 µM) > **C15** (IC_50_ = 1.29 µM; GI_50_ = 2.65 µM) > **C14** (IC_50_ = 2.20 µM; GI_50_ = 3.69 µM) > **C13** (IC_50_ = 2.73 µM; GI_50_ = 4.45 µM) ≈**C16** (IC_50_ = 2.78 µM; GI_50_ = 4.55 µM) and **C12** (IC_50_ = 0.50 µM; GI_50_ = 1.99 µM) > **C11** (IC_50_ = 1.07 µM; GI_50_ = 2.43 µM) > **C9** (IC_50_ = 1.37 µM; GI_50_ = 2.73 µM) > **C8** (IC_50_ = 2.66 µM; GI_50_ = 4.39 µM) ≈**C7** (IC_50_ = 2.60 µM; GI_50_ = 4.26 µM) > **C10** (IC_50_ = 3.24 µM; GI_50_ = 5.37 µM). As for the *para*-position only, a larger and electron-donating substitute was recommended. However, dichloro suggested electron-withdrawing substitute on *meta*-position might enhance the inhibitory activity. Thus, multi-substituted situations would be a promising direction to modify this skeleton. The data were visualized as maps and a more brief SAR analysis was displayed in the 3D QSAR part below.

### 3. Molecular Docking

**Figure 3:**
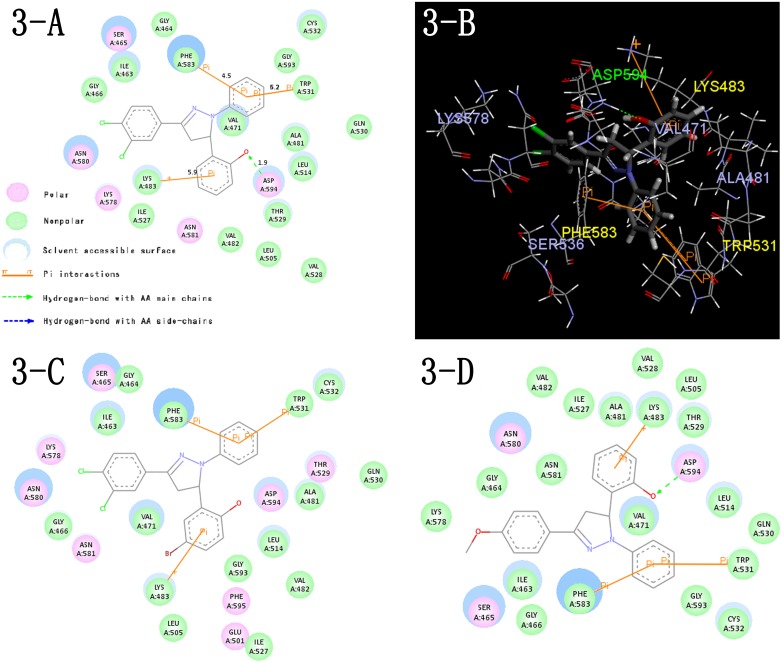
Docking models of representative compounds. (A) 2D molecular docking modeling of compound **C6** with 3PSD. (B) 3D model of the interaction between compound **C6** and 3PSD bonding site. (C) 2D molecular docking modeling of compound **C18** with 3PSD. (D) 2D molecular docking modeling of compound **C5** with 3PSD. The H-bonds (green line) are displayed as dotted lines and the amino acid they act on are labeled in green. The π–cation interactions and π–π interactions are shown as orange lines with their corresponding amino acids labeled in yellow. Other important amino acids are labeled in blue.

Molecular docking techniques were used to visualize the possible binding model of interactions between a protein (enzyme) and small molecules (ligands) [23]. In this study, the docking part was conducted using the CDOCKER protocol in Discovery Studio 3.1 (Discovery Studio 3.1, Accelrys, Inc. San Diego, CA) to visualize the probable binding method between our compounds and B-Raf. The docking of all twenty-four 2-(1,3-diaryl- 4,5-dihydro-1*H*-pyrazol-5-yl)phenol derivatives into the active site of the receptor B-Raf was performed. Two crystal structures of B-Raf (PDB Code: 3PSD.pdb [24] and 2FB8.pdb [15]) were chosen. Their original ligands were 6-[1-(piperidin-4-yl)-3-(pyridin-4-yl)-1*H*-pyrazol-4-yl]indeno[1,2-c]pyrazole (ligand code: SM7) and SB-590885, respectively. They were both obtained from the RCSB protein data bank (http://www.pdb.org). The receptor and ligands were prepared and the site sphere was chosen due to the ligand binding location. The same as another previous study [25], the results of models using 3PSD and 2FB8 were almost the same due to the generation of random conformations and the similarity of the active sites. The 2D and 3D binding maps of the most potent compound **C6** with 3PSD were depicted in Figure 3. The 2D maps of two comparisons **C18** and **C5** were also shown.

In the binding model, compound **C6** was nicely bound to 3PSD via one hydrogen bond, one π–cation interaction and several π–π interactions. The hydroxyl provided by the salicylaldehyde contributed to the hydrogen bonding interaction (O^…^H-N: 1.89 Å, 145.024°) with the amino hydrogen atom of ASP594. This might explain the advantage of introducing a hydroxyl on *ortho*-position. The mentioned π–cation interaction was formed by the same benzene ring (ring **B**) and LYS483 (distance: 5.86 Å). The π–π interactions were all formed with the new added benzene ring (ring **C**) on one end. The other ends were PHE583 (distance: 4.54 Å) and TRP531 (distance: 5.20 Å and 6.20 Å), respectively. The π–π interaction with PHE583 was exactly accordant with the previous work of B-Raf inhibitors by our group [19], [25] as well as by others [15], [24]. As for compound **C18** (2-C), the extruding effect of bulky bromo on ring **B** might disturb the formation of hydrogen bond. This might weaken the activity. As for compound **C5** (2-D), the binding pattern was similar with that of compound **C6**. The binding situations were mainly evaluated by the interactions energy. The docking calculation of all the compounds was depicted in Table 3. The CDocker Interaction Energy (interaction energy between the ligand and the receptor) agreed with the B-Raf inhibitory trend for all the synthesized compounds (linear regression: R square = 0.552, a normal level).

**Table 3:** The docking calculation of the synthesized compounds (**C1–C24**) and comparisons.

compounds	-CDOCKER INTERACTION ENERGYΔGb (kcal/mol)	compounds	-CDOCKER INTERACTION ENERGYΔGb (kcal/mol)
C1	37.0472	C13	43.1905
C2	46.3620	C14	43.5959
C3	45.9333	C15	44.5970
C4	44.3320	C16	43.1568
C5	46.3577	C17	46.1456
C6	48.6398	C18	47.1071
C7	43.2819	C19	43.5431
C8	43.2392	C20	43.6496
C9	44.4838	C21	42.9416
C10	42.8661	C22	41.3252
C11	44.9522	C23	45.0561
C12	46.3800	C24	45.1426
C0A	48.0998	C0B	47.4951

**Figure 4:**
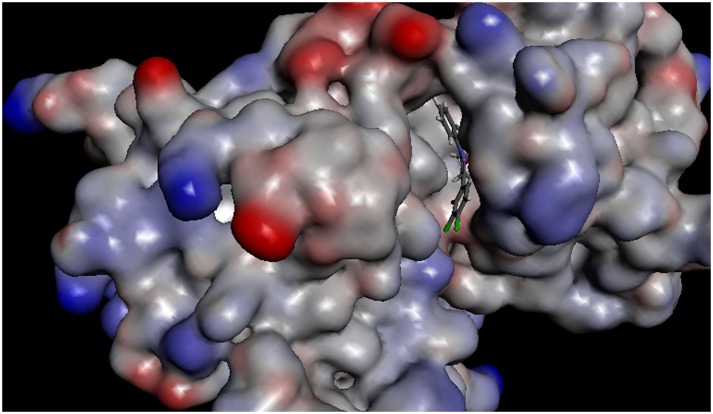
The receptor surface model with C6 in 3PSD.

The receptor surface model was shown in Figure 4, which revealed that the molecules were well embedded in the active pocket including VAL471, PHE583, ALA481, THR529, LEU514 and ASN581. This active pocket was occupied by compound **C6**, being similar as that of our previous work [19].

### 4. 3D QSAR Model

**Figure 5:**
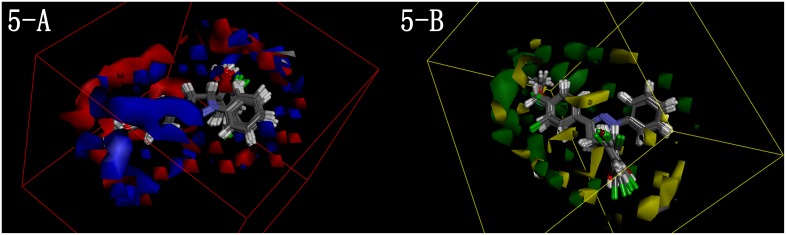
3D-QSAR of 2-(1,3-diaryl- 4,5-dihydro-1*H*-pyrazol-5-yl)phenol. Red contours mean high electron density is expected to increase activity while blue contours mean low electron density is better. Green areas mean steric bulk is better while yellow areas mean small groups are helpful.

We built a new 3D QSAR model using data of this series to check the previous one as well as to bring in the influence of hydroxyl. Using the same method as our previous work, ^19^ we utilized the Create 3D QSAR protocol of Discovery Studio 3.1 to perform the 3D QSAR of all twenty-four compounds based on the definite IC_50_ values. The values were changed into p IC_50_ scale (−log IC_50_) by convention. The training set and test set were chosen by the Diverse Molecules method in Discovery Studio 3.1. The alignment conformation of each molecule with lowest energy in the docked results of CDOCKER protocol was chosen to ensure a good alignment. The substructure 4,5-dihydro-1*H*-pyrazole was applied before building the QSAR model. The maps of 3D QSAR model were shown in Figure 5.

With the correlation coefficient r^2^ between observed activity of testing set and training set found to be 0.765, the QSAR model was proved acceptable. In Figure 5, the molecules aligned with the *iso*-surfaces of the model coefficients on electrostatic potential grids (Figure 4-A) and *Van der Waals* grids (Figure 4-B) were listed. Electrostatic map indicated regions where high electron density increase (red) or decrease (blue) activity while steric map indicated areas where steric bulk increase (green) or decrease (yellow) activity. According to the maps, the new added ring **C** (although a simple benzene ring without substitutes) enhanced the activity because slightly larger group was better there in spite of the electron situation. Meanwhile, on ring **B**, although bringing in the hydroxyl was helpful, the external situation indicated that a small and high negative charged group might be better. These points were accordant with our previous model. Finally, as for ring **A**, slightly larger substitutes would bring higher activity. A lower negative charged one was appreciated on the *para*-direction while a higher negative charged one was recommended on the *meta*-direction. Probably the introduction of multi-substitutes on ring **A** made this point a little different from our previous paper. Being in line with the previous model and the tested inhibitory activity, the 3D QSAR model provided us cogent foundation and new ideas about further design and modification.

## Conclusions

To sum up, a series of compounds (**C1–C24**) 2-(1,3-diaryl- 4,5-dihydro-1*H*-pyrazol-5-yl)phenol have been synthesized. Their B-Raf inhibitory and anti-proliferation activities were evaluated. Compound **C6** displayed the most potent biological activity against B-Raf^V600E^ and WM266.4 human melanoma cell line with corresponding IC_50_ value of 0.15 µM and GI_50_ value of 1.75 µM, being comparable with the positive controls and more potent than our previous best compounds **C0A** and **C0B**. The docking simulation was performed to get the probable binding models and poses. The results indicated that compound **C6** could bind well into the active site of B-Raf. A new 3D QSAR model was built with the activity data and binding conformations to check the previous work as well as to introduce new directions. The introduction of *ortho*-hydroxyl on 4,5-dihydro-1*H*-pyrazole skeleton did reinforce the anti-tumor activity while enlarging the group on *N*-1 of pyrazoline was also helpful.

## Methods

### 1. Chemistry

#### 1.1 General

All chemicals used were purchased from Aldrich (USA). The eluates were monitored using TLC. Melting points (uncorrected) were determined on a XT4MP apparatus (Taike Corp., Beijing, China). ESI mass spectra were obtained on a Mariner System 5304 mass spectrometer, and ^1^HNMR spectra were recorded on a DPX300 spectrometer at 25°C with TMS and solvent signals allotted as internal standards, Chemical shifts are reported in ppm (δ). Elemental analyses were performed on a CHN-O-Rapid instrument and were within 0.4% of the theoretical values. TLC was run on the silica gel coated aluminum sheets (Silica Gel 60 Å GF_254_, E. Merk, Germany) and visualized in UV light (254****nm).

#### 1.2 Compounds

**(** ***E*** **)-Chalcone** **analogues (B).** Substituted acetophenone (10 mmol) and substituted benzaldehyde (10 mmol) in ethanol (25 mL) were mixed gently at 0°C. Then 40% NaOH (5 mL) was added and stirred for 30 min. Then the mixture was placed to room temperature to continue the reaction for 4 h and the solid was filtered, washed with water and dried to obtain shiny solid **B**.

**2-(1,3-diaryl-4,5-dihydro-1H-pyrazol-5-yl)phenol (C1–C24).** **A** (5 mmol) and phenylhydrazine (5 mmol) in ethanol (20 mL) were refluxed at 80°C for 5 h. While the reaction completed, the ethanol was evaporated. The separated solid was crystallized from mixture of DMF and ethanol (9∶1) to obtain the corresponding compound as translucent solid.

**2-(3-(4-fluorophenyl)-1-phenyl-4,5-dihydro-1H-pyrazol-5-yl)phenol (C1):** White powder, yield: 57%, mp: 38–41°C. ^1^H NMR (CDCl_3_, 300 MHz) δ: 3.10–3.16 (dd, *J*_1_ = 10.5 Hz, *J*_2_ = 6.6 Hz, 1H), 3.88–3.93 (dd, *J*_1_ = 10.2 Hz, *J*_2_ = 7.5 Hz, 1H), 5.63–5.67 (m, 1H), 6.76–6.78 (t, *J* = 4.5 Hz, 1H), 6.83–6.87 (m, 2H), 6.95–6.96 (d, *J* = 4.5 Hz, 2H), 7.16–7.22 (m, 2H), 7.25–7.29 (m, 2H), 7.40–7.42 (d, J = 5.1 Hz, 2H), 7.79–7.81 (d, J = 5.4 Hz, 2H), 10.09 (m, 1H). MS (ESI): 333.13 (C_21_H_18_FN_2_O, [M+H]^+^). Anal. Calcd for C_21_H_17_FN_2_O: C, 75.89; H, 5.16; F, 5.72; N, 8.43; O, 4.81. Found: C, 75.68; H, 5.16; N, 8.44.

**2-(3-(4-chlorophenyl)-1-phenyl-4,5-dihydro-1H-pyrazol-5-yl)phenol (C2):** Yellow powder, yield: 54%, mp: 54–56°C. ^1^H NMR (CDCl_3_, 300 MHz) δ: 3.09–3.17 (dd, *J*_1_ = 10.5 Hz, *J*_2_ = 6.6 Hz, 1H), 3.87–3.93 (dd, *J*_1_ = 10.5 Hz, *J*_2_ = 7.5 Hz, 1H), 5.62–5.66 (m, 1H), 6.77–6.79 (t, *J* = 4.5 Hz, 1H), 6.84–6.88 (m, 2H), 6.97–6.98 (d, *J* = 4.8 Hz, 2H), 7.20–7.31 (m, 4H), 7.55–7.57 (d, *J* = 5.4 Hz, 2H), 7.95–7.97 (d, *J* = 5.7 Hz, 2H), 10.06 (m, 1H). MS (ESI): 349.10 (C_21_H_18_ClN_2_O, [M+H]^+^). Anal. Calcd for C_21_H_17_ClN_2_O: C, 72.31; H, 4.91; Cl, 10.16; N, 8.03; O, 4.59. Found: C, 72.13; H, 4.91; N, 8.04.

**2-(3-(4-bromophenyl)-1-phenyl-4,5-dihydro-1H-pyrazol-5-yl)phenol (C3):** Yellow powder, yield: 52%, mp: 65–68°C. ^1^H NMR (CDCl_3_, 300 MHz) δ: 3.09–3.16 (dd, *J*_1_ = 10.5 Hz, *J*_2_ = 6.6 Hz, 1H), 3.87–3.93 (dd, *J*_1_ = 10.5 Hz, *J*_2_ = 7.5 Hz, 1H), 5.61–5.65 (m, 1H), 6.76–6.78 (t, *J* = 4.8 Hz, 1H), 6.82–6.85 (m, 2H), 6.90–6.91 (d, *J* = 4.8 Hz, 2H), 7.19–7.26 (m, 4H), 7.59–7.61 (d, *J* = 5.4 Hz, 2H), 7.66–7.68 (d, *J* = 5.4 Hz, 2H), 10.04 (m, 1H). MS (ESI): 393.05 (C_21_H_18_BrN_2_O, [M+H]^+^). Anal. Calcd for C_21_H_17_BrN_2_O: C, 64.13; H, 4.36; Br, 20.32; N, 7.12; O, 4.07. Found: C, 64.01; H, 4.36; N, 7.13.

**2-(1-phenyl-3-(p-tolyl)-4,5-dihydro-1H-pyrazol-5-yl)phenol (C4):** White powder, yield: 52%, mp: 32–34°C. ^1^H NMR (CDCl_3_, 300 MHz) δ: 2.31 (s, 3H), 3.06–3.13 (dd, *J*_1_ = 10.8 Hz, *J*_2_ = 6.9 Hz, 1H), 3.84–3.91 (dd, *J*_1_ = 10.8 Hz, *J*_2_ = 7.8 Hz, 1H), 5.55–5.61 (m, 1H), 6.73–6.76 (t, *J* = 4.8 Hz, 1H), 6.81–6.87 (m, 2H), 6.91–6.93 (d, *J* = 5.1 Hz, 2H), 7.18–7.22 (m, 2H), 7.24–7.27 (m, 2H), 7.31–7.33 (d, *J* = 6.6 Hz, 2H), 7.68–7.70 (d, *J* = 5.4 Hz, 2H), 9.98 (m, 1H). MS (ESI): 329.16 (C_22_H_21_N_2_O, [M+H]^+^). Anal. Calcd for C_22_H_20_N_2_O: C, 80.46; H, 6.14; N, 8.53; O, 4.87. Found: C, 80.19; H, 6.15; N, 8.54.

**2-(3-(4-methoxyphenyl)-1-phenyl-4,5-dihydro-1H-pyrazol-5-yl)phenol (C5):** White powder, yield: 55%, mp: 35–37°C. ^1^H NMR (CDCl_3_, 300 MHz) δ: 3.05–3.12 (dd, *J*_1_ = 10.8 Hz, *J*_2_ = 6.9 Hz, 1H), 3.82 (s, 3H), 3.84–3.91 (dd, *J*_1_ = 10.8 Hz, *J*_2_ = 7.8 Hz, 1H), 5.53–5.58 (m, 1H), 6.71–6.74 (t, *J* = 4.8 Hz, 1H), 6.80–6.86 (m, 2H), 6.89–6.91 (d, *J* = 5.1 Hz, 2H), 7.10–7.13 (d, *J* = 7.2 Hz, 2H), 7.18–7.21 (m, 2H), 7.23–7.25 (m, 2H), 7.88–7.90 (d, *J* = 6.3 Hz, 2H), 9.97 (m, 1H). MS (ESI): 345.15 (C_22_H_21_N_2_O_2_, [M+H]^+^). Anal. Calcd for C_22_H_20_N_2_O_2_: C, 76.72; H, 5.85; N, 8.13; O, 9.29. Found: C, 76.51; H, 5.85; N, 8.13.

**2-(3-(3,4-dichlorophenyl)-1-phenyl-4,5-dihydro-1H-pyrazol-5-yl)phenol (C6):** Yellow powder, yield: 51%, mp: 57–59°C. ^1^H NMR (CDCl_3_, 300 MHz) δ: 3.10–3.17 (dd, *J*_1_ = 10.5 Hz, *J*_2_ = 6.3 Hz, 1H), 3.88–3.94 (dd, *J*_1_ = 10.5 Hz, *J*_2_ = 7.2 Hz, 1H), 5.64–5.67 (m, 1H), 6.78–6.81 (t, *J* = 4.2 Hz, 1H), 6.85–6.89 (m, 2H), 6.97–6.99 (m, 2H), 7.23–7.32 (m, 4H), 7.67–7.69 (d, *J* = 5.1 Hz, 1H), 7.84 (s, 1H), 7.87–7.89 (d, *J* = 5.4 Hz, 1H), 10.07 (m, 1H). MS (ESI): 383.06 (C_21_H_17_Cl_2_N_2_O, [M+H]^+^). Anal. Calcd for C_21_H_16_Cl_2_N_2_O: C, 65.81; H, 4.21; Cl, 18.50; N, 7.31; O, 4.17. Found: C, 65.63; H, 4.20; N, 7.32.

**4-chloro-2-(3-(4-fluorophenyl)-1-phenyl-4,5-dihydro-1H-pyrazol-5-yl)phenol (C7):** Yellow powder, yield: 57%, mp: 60–63°C. ^1^H NMR (CDCl_3_, 300 MHz) δ: 3.10–3.17 (dd, *J*_1_ = 10.5 Hz, *J*_2_ = 6.6 Hz, 1H), 3.89–3.95 (dd, *J*_1_ = 10.5 Hz, *J*_2_ = 7.5 Hz, 1H), 5.63–5.68 (m, 1H), 6.77–6.80 (t, *J* = 4.5 Hz, 1H), 6.95–6.99 (m, 3H), 7.15 (s, 1H), 7.21–7.23 (d, *J* = 5.1 Hz, 1H), 7.25–7.29 (m, 2H), 7.41–7.43 (d, *J* = 5.1 Hz, 2H), 7.80–7.82 (d, *J* = 5.4 Hz, 2H), 10.09 (m, 1H). MS (ESI): 367.09 (C_21_H_17_ClFN_2_O, [M+H]^+^). Anal. Calcd for C_21_H_16_ClFN_2_O: C, 68.76; H, 4.40; Cl, 9.67; F, 5.18; N, 7.64; O, 4.36. Found: C, 68.56; H, 4.40; N, 7.64.

**4-chloro-2-(3-(4-chlorophenyl)-1-phenyl-4,5-dihydro-1H-pyrazol-5-yl)phenol (C8):** Yellow powder, yield: 55%, mp: 68–71°C. ^1^H NMR (CDCl_3_, 300 MHz) δ: 3.11–3.18 (dd, *J*_1_ = 10.5 Hz, *J*_2_ = 6.6 Hz, 1H), 3.88–3.95 (dd, *J*_1_ = 10.5 Hz, *J*_2_ = 7.5 Hz, 1H), 5.62–5.66 (m, 1H), 6.78–6.80 (t, *J* = 4.5 Hz, 1H), 6.96–6.99 (m, 3H), 7.15 (s, 1H), 7.20–7.21 (d, *J* = 5.1 Hz, 1H), 7.24–7.27 (m, 2H), 7.56–7.58 (d, *J* = 5.4 Hz, 2H), 7.97–7.99 (d, *J* = 5.7 Hz, 2H), 10.11 (m, 1H). MS (ESI): 383.06 (C_21_H_17_Cl_2_N_2_O, [M+H]^+^). Anal. Calcd for C_21_H_16_Cl_2_N_2_O: C, 65.81; H, 4.21; Cl, 18.50; N, 7.31; O, 4.17. Found: C, 65.64; H, 4.21; N, 7.32.

**2-(3-(4-bromophenyl)-1-phenyl-4,5-dihydro-1H-pyrazol-5-yl)-4-chlorophenol (C9):** Yellow powder, yield: 52%, mp: 90–93°C. ^1^H NMR (CDCl_3_, 300 MHz) δ: 3.09–3.16 (dd, *J*_1_ = 10.5 Hz, *J*_2_ = 6.6 Hz, 1H), 3.87–3.94 (dd, *J*_1_ = 10.5 Hz, *J*_2_ = 7.5 Hz, 1H), 5.61–5.66 (m, 1H), 6.76–6.78 (t, *J* = 4.8 Hz, 1H), 6.93–6.97 (m, 3H), 7.13 (s, 1H), 7.18–7.19 (d, *J* = 5.4 Hz, 1H), 7.24–7.29 (m, 2H), 7.61–7.63 (d, *J* = 5.4 Hz, 2H), 7.69–7.71 (d, *J* = 5.4 Hz, 2H), 10.06 (m, 1H). MS (ESI): 427.01 (C_21_H_17_BrClN_2_O, [M+H]^+^). Anal. Calcd for C_21_H_16_BrClN_2_O: C, 58.97; H, 3.77; Br, 18.68; Cl, 8.29; N, 6.55; O, 3.74. Found: C, 58.81; H, 3.77; N, 6.56.

**4-chloro-2-(1-phenyl-3-(p-tolyl)-4,5-dihydro-1H-pyrazol-5-yl)phenol (C10):** White powder, yield: 54%, mp: 43–46°C. ^1^H NMR (CDCl_3_, 300 MHz) δ: 2.33 (s, 3H), 3.07–3.14 (dd, *J*_1_ = 10.8 Hz, *J*_2_ = 6.9 Hz, 1H), 3.85–3.92 (dd, *J*_1_ = 10.8 Hz, *J*_2_ = 7.8 Hz, 1H), 5.57–5.64 (m, 1H), 6.74–6.77 (t, *J* = 4.8 Hz, 1H), 6.92–6.95 (m, 3H), 7.12 (s, 1H), 7.16–7.18 (d, *J* = 5.4 Hz, 1H), 7.23–7.27 (m, 2H), 7.31–7.33 (d, *J* = 6.6 Hz, 2H), 7.69–7.71 (d, *J* = 5.4 Hz, 2H), 10.01 (m, 1H). MS (ESI): 363.12 (C_22_H_20_ClN_2_O, [M+H]^+^). Anal. Calcd for C_22_H_19_ClN_2_O: C, 72.82; H, 5.28; Cl, 9.77; N, 7.72; O, 4.41. Found: C, 72.60; H, 5.28; N, 7.73.

**4-chloro-2-(3-(4-methoxyphenyl)-1-phenyl-4,5-dihydro-1H-pyrazol-5-yl)phenol (C11):** White powder, yield: 56%, mp: 49–51°C. ^1^H NMR (CDCl_3_, 300 MHz) δ: 3.06–3.13 (dd, *J*_1_ = 10.8 Hz, *J*_2_ = 6.9 Hz, 1H), 3.82 (s, 3H), 3.85–3.91 (dd, *J*_1_ = 10.8 Hz, *J*_2_ = 7.8 Hz, 1H), 5.56–5.62 (m, 1H), 6.73–6.76 (t, *J* = 4.8 Hz, 1H), 6.90–6.94 (m, 3H), 7.11 (s, 1H), 7.11–7.14 (d, *J* = 7.2 Hz, 2H), 7.15–7.17 (d, *J* = 5.4 Hz, 1H), 7.23–7.26 (m, 2H), 7.89–7.91 (d, *J* = 6.3 Hz, 2H), 9.99 (m, 1H). MS (ESI): 379.11 (C_22_H_20_ClN_2_O_2_, [M+H]^+^). Anal. Calcd for C_22_H_19_ClN_2_O_2_: C, 69.75; H, 5.05; Cl, 9.36; N, 7.39; O, 8.45. Found: C, 69.52; H, 5.05; N, 7.40.

**4-chloro-2-(3-(3,4-dichlorophenyl)-1-phenyl-4,5-dihydro-1H-pyrazol-5-yl)phenol (C12):** Yellow powder, yield: 51%, mp: 77–79°C. ^1^H NMR (CDCl_3_, 300 MHz) δ: 3.12–3.18 (dd, *J*_1_ = 10.5 Hz, *J*_2_ = 6.0 Hz, 1H), 3.88–3.94 (dd, *J*_1_ = 10.5 Hz, *J*_2_ = 7.2 Hz, 1H), 5.64–5.67 (m, 1H), 6.79–6.81 (t, *J* = 4.2 Hz, 1H), 6.97–7.01 (m, 3H), 7.17 (s, 1H), 7.23–7.24 (d, *J* = 4.8 Hz, 1H), 7.27–7.29 (t, *J* = 4.2 Hz, 2H), 7.69–7.71 (d, *J* = 4.8 Hz, 1H), 7.85–7.88 (m, 2H), 10.12 (m, 1H). MS (ESI): 417.02 (C_21_H_16_Cl_3_N_2_O, [M+H]^+^). Anal. Calcd for C_21_H_15_Cl_3_N_2_O: C, 60.38; H, 3.62; Cl, 25.46; N, 6.71; O, 3.83. Found: C, 60.19; H, 3.62; N, 6.72.

**4-bromo-2-(3-(4-fluorophenyl)-1-phenyl-4,5-dihydro-1H-pyrazol-5-yl)phenol (C13):** Yellow powder, yield: 51%, mp: 73–76°C. ^1^H NMR (CDCl_3_, 300 MHz) δ: 3.10–3.17 (dd, *J*_1_ = 10.5 Hz, *J*_2_ = 6.6 Hz, 1H), 3.88–3.94 (dd, *J*_1_ = 10.2 Hz, *J*_2_ = 7.2 Hz, 1H), 5.63–5.67 (m, 1H), 6.76–6.79 (t, *J* = 4.5 Hz, 1H), 6.91–6.97 (m, 3H), 7.08 (s, 1H), 7.21–7.23 (m, 2H), 7.31–7.33 (d, *J* = 5.4 Hz, 1H), 7.40–7.42 (d, *J* = 5.4 Hz, 2H), 7.79–7.81 (d, *J* = 5.4 Hz, 2H), 10.07 (m, 1H). MS (ESI): 411.04 (C_21_H_17_BrFN_2_O, [M+H]^+^). Anal. Calcd for C_21_H_16_BrFN_2_O: C, 61.33; H, 3.92; Br, 19.43; F, 4.62; N, 6.81; O, 3.89. Found: C, 61.11; H, 3.92; N, 6.81.

**4-bromo-2-(3-(4-chlorophenyl)-1-phenyl-4,5-dihydro-1H-pyrazol-5-yl)phenol (C14):** Yellow powder, yield: 53%, mp: 88–91°C. ^1^H NMR (CDCl_3_, 300 MHz) δ: 3.10–3.16 (dd, *J*_1_ = 10.5 Hz, *J*_2_ = 6.6 Hz, 1H), 3.87–3.94 (dd, *J*_1_ = 10.5 Hz, *J*_2_ = 7.5 Hz, 1H), 5.62–5.67 (m, 1H), 6.77–6.79 (t, *J* = 4.5 Hz, 1H), 6.90–6.96 (m, 3H), 7.07 (s, 1H), 7.20–7.24 (m, 2H), 7.30–7.33 (d, *J* = 5.4 Hz, 1H), 7.55–7.57 (d, *J* = 5.4 Hz, 2H), 7.95–7.98 (d, *J* = 5.7 Hz, 2H), 10.05 (m, 1H). MS (ESI): 427.01 (C_21_H_17_BrClN_2_O, [M+H]^+^). Anal. Calcd for C_21_H_16_BrClN_2_O: C, 58.97; H, 3.77; Br, 18.68; Cl, 8.29; N, 6.55; O, 3.74. Found: C, 58.78; H, 3.78; N, 6.56.

**4-bromo-2-(3-(4-bromophenyl)-1-phenyl-4,5-dihydro-1H-pyrazol-5-yl)phenol (C15):** Yellow powder, yield: 52%, mp: 104–106°C. ^1^H NMR (CDCl_3_, 300 MHz) δ: 3.09–3.16 (dd, *J*_1_ = 10.5 Hz, *J*_2_ = 6.6 Hz, 1H), 3.87–3.93 (dd, *J*_1_ = 10.5 Hz, *J*_2_ = 7.5 Hz, 1H), 5.61–5.65 (m, 1H), 6.76–6.78 (t, *J* = 4.5 Hz, 1H), 6.90–6.95 (m, 3H), 7.06 (s, 1H), 7.19–7.23 (m, 2H), 7.30–7.32 (d, *J* = 5.4 Hz, 1H), 7.60–7.62 (d, *J* = 5.4 Hz, 2H), 7.67–7.69 (d, *J* = 5.4 Hz, 2H), 10.05 (m, 1H). MS (ESI): 470.96 (C_21_H_17_Br_2_N_2_O, [M+H]^+^). Anal. Calcd for C_21_H_16_Br_2_N_2_O: C, 53.42; H, 3.42; Br, 33.85; N, 5.93; O, 3.39. Found: C, 53.30; H, 3.42; N, 5.93.

**4-bromo-2-(1-phenyl-3-(p-tolyl)-4,5-dihydro-1H-pyrazol-5-yl)phenol (C16):** Yellow powder, yield: 51%, mp: 59–63°C. ^1^H NMR (CDCl_3_, 300 MHz) δ: 2.33 (s, 3H), 3.06–3.13 (dd, *J*_1_ = 10.8 Hz, *J*_2_ = 6.9 Hz, 1H), 3.84–3.91 (dd, *J*_1_ = 10.8 Hz, *J*_2_ = 7.8 Hz, 1H), 5.55–5.60 (m, 1H), 6.73–6.75 (t, *J* = 4.8 Hz, 1H), 6.85–6.91 (m, 3H), 7.07 (s, 1H), 7.20–7.24 (m, 2H), 7.29–7.34 (m, 3H), 7.68–7.70 (d, *J* = 5.4 Hz, 2H), 10.00 (m, 1H). MS (ESI): 407.07 (C_22_H_20_BrN_2_O, [M+H]^+^). Anal. Calcd for C_22_H_19_BrN_2_O: C, 64.87; H, 4.70; Br, 19.62; N, 6.88; O, 3.93. Found: C, 64.71; H, 4.70; N, 6.89.

**4-bromo-2-(3-(4-methoxyphenyl)-1-phenyl-4,5-dihydro-1H-pyrazol-5-yl)phenol (C17):** Yellow powder, yield: 56%, mp: 65–67°C. ^1^H NMR (CDCl_3_, 300 MHz) δ: 3.05–3.12 (dd, *J*_1_ = 10.8 Hz, *J*_2_ = 6.9 Hz, 1H), 3.82 (s, 3H), 3.84–3.91 (dd, *J*_1_ = 10.8 Hz, *J*_2_ = 7.8 Hz, 1H), 5.54–5.60 (m, 1H), 6.72–6.74 (t, *J* = 4.8 Hz, 1H), 6.83–6.90 (m, 3H), 7.05 (s, 1H), 7.10–7.13 (d, *J* = 7.2 Hz, 2H), 7.18–7.22 (m, 2H), 7.34–7.35 (d, *J* = 5.4 Hz, 1H), 7.89–7.91 (d, *J* = 6.3 Hz, 2H), 9.96 (m, 1H). MS (ESI): 423.06 (C_22_H_20_BrN_2_O_2_, [M+H]^+^). Anal. Calcd for C_22_H_19_BrN_2_O_2_: C, 62.42; H, 4.52; Br, 18.88; N, 6.62; O, 7.56. Found: C, 62.29; H, 4.52; N, 6.63.

**4-bromo-2-(3-(3,4-dichlorophenyl)-1-phenyl-4,5-dihydro-1H-pyrazol-5-yl)phenol (C18):** Yellow powder, yield: 53%, mp: 95–98°C. ^1^H NMR (CDCl_3_, 300 MHz) δ: 3.11–3.16 (dd, *J*_1_ = 10.5 Hz, *J*_2_ = 6.3 Hz, 1H), 3.88–3.95 (dd, *J*_1_ = 10.5 Hz, *J*_2_ = 7.2 Hz, 1H), 5.63–5.67 (m, 1H), 6.79–6.81 (t, *J* = 4.2 Hz, 1H), 6.91–6.97 (m, 3H), 7.09 (s, 1H), 7.22–7.27 (m, 2H), 7.31–7.34 (d, *J* = 5.1 Hz, 1H), 7.67–7.69 (d, *J* = 4.8 Hz, 1H), 7.83 (s, 1H), 7.85–7.87 (d, *J* = 5.1 Hz, 1H), 10.06 (m, 1H). MS (ESI): 460.97 (C_21_H_16_BrCl_2_N_2_O, [M+H]^+^). Anal. Calcd for C_21_H_15_BrCl_2_N_2_O: C, 54.57; H, 3.27; Br, 17.29; Cl, 15.34; N, 6.06; O, 3.46. Found: C, 54.45; H, 3.27; N, 6.06.

**2,4-dichloro-6-(3-(4-fluorophenyl)-1-phenyl-4,5-dihydro-1H-pyrazol-5-yl)phenol (C19):** Yellow powder, yield: 54%, mp: 37–39°C. ^1^H NMR (CDCl_3_, 300 MHz) δ: 3.09–3.13 (dd, *J*_1_ = 10.8 Hz, *J*_2_ = 4.2 Hz, 1H), 3.83–3.90 (dd, *J*_1_ = 11.4 Hz, *J*_2_ = 5.7 Hz, 1H), 5.63–5.67 (m, 1H), 6.77–6.81 (t, *J* = 4.2 Hz, 1H), 6.86 (s, 1H), 6.97–6.99 (d, *J* = 4.8 Hz, 2H), 7.20–7.24 (t, *J* = 4.8 Hz, 2H), 7.38–7.40 (d, *J* = 4.8 Hz, 2H), 7.44 (s, 1H), 7.78–7.80 (d, *J* = 5.4 Hz, 2H), 10.07 (m, 1H). MS (ESI): 401.05 (C_21_H_16_Cl_2_FN_2_O, [M+H]^+^). Anal. Calcd for C_21_H_15_Cl_2_FN_2_O: C, 62.86; H, 3.77; Cl, 17.67; F, 4.73; N, 6.98; O, 3.99. Found: C, 62.63; H, 3.76; N, 6.99.

**2,4-dichloro-6-(3-(4-chlorophenyl)-1-phenyl-4,5-dihydro-1H-pyrazol-5-yl)phenol (C20):** Yellow powder, yield: 53%, mp: 42–43°C. ^1^H NMR (CDCl_3_, 300 MHz) δ: 3.08–3.12 (dd, *J*_1_ = 10.2 Hz, *J*_2_ = 4.2 Hz, 1H), 3.83–3.89 (dd, *J*_1_ = 11.7 Hz, *J*_2_ = 5.7 Hz, 1H), 5.65–5.69 (dd, *J*_1_ = 7.2 Hz, *J*_2_ = 3.9 Hz, 1H), 6.77–6.80 (t, *J* = 4.2 Hz, 1H), 6.86 (s, 1H), 6.96–6.98 (d, *J* = 4.5 Hz, 2H), 7.21–7.24 (t, *J* = 4.5 Hz, 2H), 7.43 (s, 1H), 7.55–7.57 (d, *J* = 5.1 Hz, 2H), 7.96–7.98 (d, *J* = 5.4 Hz, 2H), 10.08 (m, 1H). MS (ESI): 417.02 (C_21_H_16_Cl_3_N_2_O, [M+H]^+^). Anal. Calcd for C_21_H_15_Cl_3_N_2_O: C, 60.38; H, 3.62; Cl, 25.46; N, 6.71; O, 3.83. Found: C, 60.23; H, 3.62; N, 6.71.

**2-(3-(4-bromophenyl)-1-phenyl-4,5-dihydro-1H-pyrazol-5-yl)-4,6-dichlorophenol (C21):** Yellow powder, yield: 51%, mp: 56–59°C. ^1^H NMR (CDCl_3_, 300 MHz) δ: 3.08–3.13 (dd, *J*_1_ = 10.5 Hz, *J*_2_ = 3.9 Hz, 1H), 3.82–3.89 (dd, *J*_1_ = 11.7 Hz, *J*_2_ = 5.4 Hz, 1H), 5.63–5.67 (dd, *J*_1_ = 7.5 Hz, *J*_2_ = 3.9 Hz, 1H), 6.76–6.79 (t, *J* = 4.2 Hz, 1H), 6.84 (s, 1H), 6.95–6.96 (d, *J* = 4.8 Hz, 2H), 7.19–7.22 (t, *J* = 4.8 Hz, 2H), 7.41–7.42 (m, 1H), 7.60–7.62 (d, *J* = 5.4 Hz, 2H), 7.67–7.69 (d, *J* = 5.1 Hz, 2H), 10.10 (m, 1H). MS (ESI): 460.97 (C_21_H_16_BrCl_2_N_2_O, [M+H]^+^). Anal. Calcd for C_21_H_15_BrCl_2_N_2_O: C, 54.57; H, 3.27; Br, 17.29; Cl, 15.34; N, 6.06; O, 3.46. Found: C, 54.49; H, 3.26; N, 6.07.

**2,4-dichloro-6-(1-phenyl-3-(p-tolyl)-4,5-dihydro-1H-pyrazol-5-yl)phenol (C22):** Yellow powder, yield: 53%, mp: 29–31°C. ^1^H NMR (CDCl_3_, 300 MHz) δ: 2.34 (s, 3H), 3.06–3.11 (dd, *J*_1_ = 11.4 Hz, *J*_2_ = 4.5 Hz, 1H), 3.81–3.88 (dd, *J*_1_ = 12.0 Hz, *J*_2_ = 5.7 Hz, 1H), 5.61–5.65 (m, 1H), 6.74–6.78 (t, *J* = 4.5 Hz, 1H), 6.82 (s, 1H), 6.93–6.95 (d, *J* = 4.8 Hz, 2H), 7.18–7.21 (t, *J* = 4.8 Hz, 2H), 7.30–7.32 (d, *J* = 5.7 Hz, 2H), 7.39–7.41 (m, 1H), 7.69–7.71 (d, *J* = 5.1 Hz, 2H), 10.03 (m, 1H). MS (ESI): 397.08 (C_22_H_19_Cl_2_N_2_O, [M+H]^+^). Anal. Calcd for C_22_H_18_Cl_2_N_2_O: C, 66.51; H, 4.57; Cl, 17.85; N, 7.05; O, 4.03. Found: C, 66.37; H, 4.57; N, 7.06.

**2,4-dichloro-6-(3-(4-methoxyphenyl)-1-phenyl-4,5-dihydro-1H-pyrazol-5-yl)phenol (C23):** Yellow powder, yield: 55%, mp: 33–34°C. ^1^H NMR (CDCl_3_, 300 MHz) δ: 3.05–3.12 (dd, *J*_1_ = 12.0 Hz, *J*_2_ = 4.5 Hz, 1H), 3.82 (s, 3H), 3.83–3.90 (dd, *J*_1_ = 12.0 Hz, *J*_2_ = 5.7 Hz, 1H), 5.59–5.65 (m, 1H), 6.73–6.78 (t, *J* = 4.5 Hz, 1H), 6.81 (s, 1H), 6.93–6.95 (d, *J* = 4.8 Hz, 2H), 7.08–7.10 (d, *J* = 5.7 Hz, 2H), 7.17–7.21 (t, *J* = 4.8 Hz, 2H), 7.39 (s, 1H), 7.88–7.90 (d, *J* = 5.1 Hz, 2H), 10.03 (m, 1H). MS (ESI): 413.07 (C_22_H_19_Cl_2_N_2_O_2_, [M+H]^+^). Anal. Calcd for C_22_H_18_Cl_2_N_2_O_2_: C, 63.93; H, 4.39; Cl, 17.16; N, 6.78; O,7.74. Found: C, 63.77; H, 4.38; N, 6.79.

**2,4-dichloro-6-(3-(3,4-dichlorophenyl)-1-phenyl-4,5-dihydro-1H-pyrazol-5-yl)phenol (C24):** Yellow powder, yield: 55%, mp: 63–65°C. ^1^H NMR (CDCl_3_, 300 MHz) δ: 3.10–3.14 (dd, *J*_1_ = 10.2 Hz, *J*_2_ = 4.2 Hz, 1H), 3.84–3.89 (dd, *J*_1_ = 11.1 Hz, *J*_2_ = 5.4 Hz, 1H), 5.67–5.70 (m, 1H), 6.78–6.81 (t, *J* = 4.2 Hz, 1H), 6.88 (s, 1H), 6.98–7.00 (d, *J* = 4.2 Hz, 2H), 7.23–7.26 (t, *J* = 4.2 Hz, 2H), 7.44 (s, 1H), 7.71–7.73 (d, *J* = 4.8 Hz, 1H), 7.84–7.87 (m, 2H), 10.08 (m, 1H). MS (ESI): 450.99 (C_21_H_15_Cl_4_N_2_O, [M+H]^+^). Anal. Calcd for C_21_H_14_Cl_4_N_2_O: C, 55.78; H, 3.12; Cl, 31.36; N, 6.20; O,3.54. Found: C, 55.61; H, 3.12; N, 6.21.

### 2. Biological Assay

#### 2.1 Anti-proliferation assay

WM266.4 melanoma cells [22] were cultured in DMEM/10% fetal bovine serum, in 5% CO_2_ water saturated atmosphere at 37°C. Cell suspensions (10000/mL) were prepared and 100** µ**L/well dispensed into 96-well plates (Costar) giving 1000 cells/well. The plates were returned to the incubator for 24****h to allow the cells to reattach. These compounds were initially prepared at 20****mM in DMSO. Aliquots (200** µ**L) were diluted into 20****mL culture medium giving 200** µ**M, and 10 serial dilutions of 3x prepared. Aliquots (100** µ**L) of each dilution were added to the wells, giving doses ranging from 100** µ**M to 0.005** µ**M. After a further incubated at 37°C for 24****h in a humidified atmosphere with 5% CO_2_, the cell viability was assessed by the conventional 3-(4,5-dimethylthiazol-2-yl)-2,5-diphenyltetrazolium bromide (MTT) reduction assay and carried out strictly according to the manufacturer instructions (Sigma). The absorbance at 590****nm was recorded using LX300 Epson Diagnostic micro-plate reader. Then GI_50_ was calculated using SPSS 13.0 software.

#### 2.2 Kinase inhibitory assay

This V600E mutant B-Raf kinase assay was performed in triplicate for each tested compound in this study. Briefly, 7.5 ng Mouse Full-Length GST-tagged BRAF^V600E^ (Invitrogen, PV3849) was preincubated at room temperature for 1 h with 1 µL drug and 4 µL assay dilution buffer. The kinase assay was initiated when 5 µL of a solution containing 200 ng recombinant human full length, N-terminal His-tagged MEK1 (Invitrogen), 200 µM ATP, and 30 mM MgCl_2_ in assay dilution buffer was added. The kinase reaction was allowed to continue at room temperature for 25 min and was then quenched with 5 µL 5x protein denaturing buffer (LDS) solution. Protein was further denatured by heating for 5 min at 70°C. 10 µL of each reaction was loaded into a 15-well, 4–12% precast NuPage gel (Invitrogen) and run at 200 V, and upon completion, the front, which contained excess hot ATP, was cut from the gel and discarded. The gel was then dried and developed onto a phosphor screen. A reaction that contained no active enzyme was used as a negative control, and a reaction without inhibitor was used as the positive control.

Detection of the effect of compounds on cell based pERK1/2 activity in WM266.4 cells was performed using ELISA kits (Invitrogen) and strictly according to the manufacturer instructions.

### 3. Experimental Protocol of Docking Study

The three-dimensional structures of the aforementioned compounds were constructed using Chem. 3D ultra 12.0 software [Chemical Structure Drawing Standard; Cambridge Soft corporation, USA (2010)], then they were energetically minimized by using MMFF94 with 5000 iterations and minimum RMS gradient of 0.10. The crystal structures of B-Raf kinase domain bound to SB-590885 (PDB code: 2FB8) and bound to SM7 (PDB code: 3PSD) complex were retrieved from the RCSB Protein Data Bank (http://www.rcsb.org/pdb/home/home.do). All bound waters and ligands were eliminated from the protein and the polar hydrogen was added to the proteins. Molecular docking of all twenty-four compounds as well as **C0A** and **C0B** was then carried out using the Discovery Stutio (version 3.1) as implemented through the graphical user interface CDocker protocal.

CDOCKER is an implementation of a CHARMm based molecular docking tool using a half-flexible receptor [26], including the following steps:

1. A series of ligands conformations are generated using high temperature molecular dynamics with different random seeds.
2. Random orientations of the conformations are generated by translating the center of the ligand to a specified position within the receptor active site, and making a series of random rotations. A softened energy is calculated and the orientation is kept when it is less than a specified limit. This process repeats until either the desired number of low-energy orientations is obtained, or the test times of bad orientations reached the maximum number.
3. Each orientation is subjected to simulated annealing molecular dynamics. The temperature is heated up to a high temperature then cooled to the target temperature. A final energy minimization of the ligand in the rigid receptor using non-softened potential is performed.
4. For each of the final pose, the CHARMm energy (interaction energy plus ligand strain) and the interaction energy alone are figured out. The poses are sorted according to CHARMm energy and the top scoring (most negative, thus favorable to binding) poses are retained. The whole B-Raf kinase domain defined as a receptor and the site sphere was selected based on the original ligand binding location, then the original ligand was removed and the ligands prepared by us were placed during the molecular docking procedure. CHARMm was selected as the force field. The molecular docking was performed with a simulated annealing method. The heating steps were 2000 with 700 of heating target temperature. The cooling steps were 5000 with 300 cooling target temperature. Ten molecular docking poses saved for each ligand were ranked according to their dock score function. The pose with the highest -CDOCKER energy was chosen as the most suitable pose.

### 4. Experimental Protocol of QSAR Model

Among all the 24 compounds, 87.5% (that is 21) were utilized as a training set for QSAR modeling. The remaining 12.5% (that is 3) were chosen as an external test subset for validating the reliability of the QSAR model by the Diverse Molecules protocol in Discovery Studio 3.1. The selected test compounds were: **C5**, **C8**, **C15**.

The inhibitory activity of the compounds in literatures [IC_50_ (mol/L)] was initially changed into the minus logarithmic scale [p IC_50_ (mol/L)] and then used for subsequent QSAR analysis as the response variable.

In Discovery Studio, the CHARMm force field is applied and the electrostatic potential together with the *Van der Waals* potential are treated as separate terms. As the electrostatic potential probe, A+le point change is used while distance-dependent dielectric constant is used to mimic the solvent effect. As for the Van der Waals potential, a carbon atom with a radius of 1.73 Å is used as a probe.

A Partial Least-Squares (PLS) model is built using energy grids as descriptors. QSAR models were built by using the Create 3D QSAR Model protocol in Discovery Studio 3.1.

## References

[1] El-Azab AS, Al-Omar MA, Abdel-Aziz AM, Abdel-Aziz NI, El-Sayed AA, et al (2010) Design, synthesis and biological evaluation of novel quinazoline derivatives as potential antitumor agents: Molecular docking study. Eur J Med Chem 45: 4188–4198. 20599299 10.1016/j.ejmech.2010.06.013

[2] Wellbrock C, Karasarides M, Marais R (2004) The RAF proteins take centre stage. Nat Rev Mol Cell Biol 5: 875–885. 15520807 10.1038/nrm1498

[3] Li N, Batt D, Warmuth M (2007) B-Raf kinase inhibitors for cancer treatment. Curr Opin Invest Drugs 8: 452–456. 17621874

[4] Hoshino R, Chantani Y, Yamori T, Tsuruo T, Oka H, et al (1999) Constitutive activation of the 41-/43-kDa mitogen-activated protein kinase signaling pathway in human tumors. Oncogene 18: 813–822. 9989833 10.1038/sj.onc.1202367

[5] Davies H, Bignell GR, Cox C, Stephens P, Edkins S, et al (2002) Mutations of the BRAF gene in human cancer. Nature 417: 949–954. 12068308 10.1038/nature00766

[6] Cohen Y, Xing M, Mambo E, Guo Z, Wu G, et al (2003) BRAF Mutation in papillary thyroid carcinoma. J Natl Cancer Inst 95: 625–627. 12697856 10.1093/jnci/95.8.625

[7] Xu X, Quiros RM, Gattuso P, Ain KB, Prinz RA (2003) High prevalence of BRAF gene mutation in papillary thyroid carcinomas and thyroid tumor cell lines. Canc Res 63: 4561–4567. 12907632

[8] Kalinsky K, Haluska FG (2007) Novel inhibitors in the treatment of metastatic melanoma. Expert Rev Anticancer Ther 7: 715–724. 17492934 10.1586/14737140.7.5.715

[9] Dhillon AS, Hagan S, Rath O, Kolch W (2007) MAP kinase signalling pathways in cancer. Oncogene 26: 3279–3290. 17496922 10.1038/sj.onc.1210421

[10] Wan PT, Garnet MJ, Roe SM, Lee S, Niculescu-Duvaz D, et al (2004) Mechanism of activation of the RAF-ERK signaling pathway by oncogenic mutations of B-RAF. Cell 116: 855–867. 15035987 10.1016/s0092-8674(04)00215-6

[11] Gray-Schopfer VC, Dias SD, Marais R (2005) Erratum, the role of B-RAF in melanoma (vol 24, 1, 2005). Cancer Metast Rev 24: 367–367. 10.1007/s10555-005-5865-1 15785879

[12] Sommers JA, Sharma S, Doherty KM, Karmakar P, Yang Q, et al (2005) P53 Modulates RPA-dependent and RPA-independent WRN helicase activity. Canc Res 65: 1223–1233. 10.1158/0008-5472.CAN-03-0231 15735006

[13] Karasarides M, Chiloeches A, Hayward R, Niculescu-Duvaz D, Scanlon I, et al (2004) B-RAF is a therapeutic target in melanoma. Oncogene 23: 6292–6298. 15208680 10.1038/sj.onc.1207785

[14] Mathieu S, Gradl SN, Ren L, Wen Z, Aliagas I, et al (2012) Potent and Selective Aminopyrimidine-Based B-Raf Inhibitors with Favorable Physicochemical and Pharmacokinetic Properties. J Med Chem 55: 2869–2881. 22335519 10.1021/jm300016v

[15] King AJ, Patrick DR, Batorsky RS, Ho ML, Do HT, et al (2006) Demonstration of a genetic therapeutic index for tumors expressing oncogenic BRAF by the kinase inhibitor SB-590885. Canc Res 66: 11100–11105. 10.1158/0008-5472.CAN-06-2554 17145850

[16] De DYD, Krogstad FM, Byers LD, Krogstad DJ (1998) Structure-activity relationships for antiplasmodial activity among 7-substituted 4-aminoquinolines. J Med Chem 41: 4918–4926. 9836608 10.1021/jm980146x

[17] Azarifar D, Ghasemnejad H (2003) Microwave-assisted synthesis of some 3,5-arylated 2-pyrazolines. Molecules 8: 642–648.

[18] Blackburn C, Duffy MO, Gould AE, Kulkarni B, Liu JX, et al (2010) Discovery and optimization of *N*-acyl and *N*-aroylpyrazolines as B-Raf kinase inhibitors. Bioorg Med Chem Lett 20: 4795–4799. 20630752 10.1016/j.bmcl.2010.06.110

[19] Li CY, Li QS, Yan Li, Sun XG, Wei R, et al (2012) Synthesis, biological evaluation and 3D-QSAR studies of novel 4,5-dihydro-1*H*-pyrazole niacinamide derivatives as BRAF inhibitors. Bioorg Med Chem 20: 3746–3755. 22583669 10.1016/j.bmc.2012.04.047

[20] Liu JJ, Zhang H, Sun J, Wang ZC, Yang YS, et al (2012) Synthesis, biological evaluation of novel 4,5-dihydro-2*H*-pyrazole 2-hydroxyphenyl derivatives as BRAF inhibitors. Bioorg Med Chem 20: 6089–6096. 22985957 10.1016/j.bmc.2012.08.020

[21] Latif N, Abdel Meguid S, Swellem RH (1983) Newer halo- and hydroxyaryl pyrazolines and -pyrazoles and their infrared spectra. Egyptian J Chem 26: 521–7.

[22] Niculescu-Duvaz D, Niculescu-Duvaz I, Suijkerbuijk B, Ménard D, Zambon A, et al (2010) Novel tricyclic pyrazole BRAF inhibitors with imidazole or furan central scaffolds. Bioorg Med Chem 18: 6934–6952. 20667740 10.1016/j.bmc.2010.06.031 PMC2956513

[23] Kirkpatrick P (2004) Virtual screening - Gliding to success. Nat Rev Drug Discov 3: 299.

[24] Newhouse BJ, Hansen JD, Grina J, Welch M, Topalov G, et al (2011) Non-oxime pyrazole based inhibitors of B-Raf kinase. Bioorg Med Chem Lett 21: 3488–3492. 21536432 10.1016/j.bmcl.2010.12.038

[25] Yang YS, Li QS, Sun S, Zhang YB, Wang XL, et al (2012) Design, modification and 3D QSAR studies of novel 2,3-dihydrobenzo[*b*][1,4]dioxin-containing 4,5-dihydro-1*H*-pyrazole derivatives as inhibitors of B-Raf kinase. Bioorg Med Chem 20: 6048–6058. 22985962 10.1016/j.bmc.2012.08.043

[26] Wu GS, Robertson DH, Brooks CL, Vieth M (2003) Detailed analysis of grid-based molecular docking: A case study of CDOCKER - A CHARMm-based MD docking algorithm. J Comput Chem 24: 1549–1562. 12925999 10.1002/jcc.10306

